# Lubrication by plant-based emulsions: Linking oil-water protein-stabilized interfacial mechanical properties to oil droplet lubrication properties

**DOI:** 10.1016/j.crfs.2025.101270

**Published:** 2025-12-06

**Authors:** Lei Ji, Leonard M.C. Sagis, Elke Scholten, Jack Yang

**Affiliations:** Wageningen University, Agrotechnology & Food Sciences Group, Laboratory of Physics and Physical Chemistry of Foods, P.O. Box 17, Wageningen, 6700 AA, the Netherlands

**Keywords:** Tribology, Interfacial dilatational rheology, Protein emulsions, Oil-water interface, Plant protein, General stress decomposition

## Abstract

This work investigates how the viscoelasticity of the protein layer at the oil-water interface of emulsion droplets governs the emulsion lubrication behavior. Commercially-available (PPIC) and lab-produced (PPIL) pea protein isolate, and soy protein isolate (SPI), were used to stabilize the emulsions. Whey protein isolate (WPI) served as a reference system. We found that WPI formed stiff, solid-like interfacial layers, and PPIL formed an interface that exhibits high deformability. Both interfaces were strong enough to resist mechanical stresses. In contrast, PPIC and SPI were heavily aggregated in bulk solution, forming much weaker oil-water interfaces, which were disrupted at higher stresses. The emulsion droplets stabilized by WPI or PPIL remained stable under mechanical stress, and the oil droplets were hypothesized to act as particles that limited contact between the interacting surfaces, thereby providing lubrication via a rolling/sliding mechanism. In contrast, the PPIC- and SPI-stabilized emulsions exhibited more effective friction reduction, which was hypothesized to result from oil droplet coalescence and the subsequent formation of a lubricating film. These lubrication behaviors showed a high correlation with the mechanical properties of oil-water interfaces stabilized by the proteins, i.e. elastic dilatational moduli (*Ed'* and *Ed''*) and viscous dissipation of the odd (U_dτ2_) and even (U_dτ3_) harmonics. These results show that protein oil-water interfacial properties, especially the mobility and resistance against density change of adsorbed proteins, are strongly correlated with lubrication properties, indicating that by structuring the oil-water interface with certain proteins, lubrication properties can be achieved, offering a strategy to tailor mouthfeel.

## Highlights

• Stiff or deformable protein interfacial layers lead to particle-based lubrication.
• Weak interfacial films rupture and the oil leakage give film-based lubrication.
• The type of interface formed can be linked the protein molecular properties.
• Interfacial properties showed high correlation with lubrication properties.

## Graphical abstract

**Figure:**
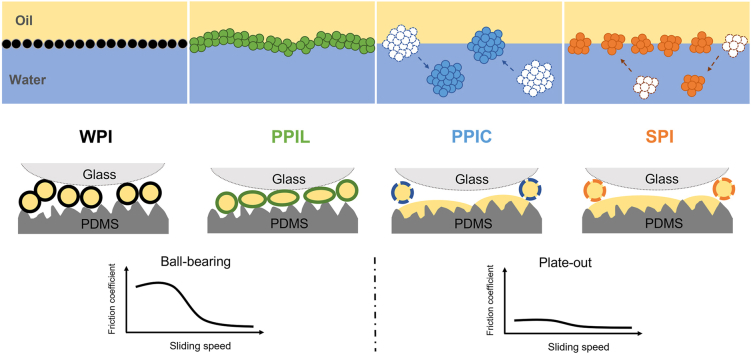

## 1 Introduction

The growing popularity of plant-based foods has increased the need for emulsifiers derived from plant sources. Emulsifiers are essential for stabilizing emulsions and are widely used in the food industry to develop a variety of different food products, based on emulsions. A key challenge in this area is the limited understanding of the mechanical stability of emulsion droplets and underlying mechanisms, which directly affect the structure of the food matrix and, consequently the texture and physical stability of foods. These physical characteristics play an important role in determining lubrication properties (Dresselhuis et al., 2008; Ji et al., 2024), which is closely linked to mouthfeel, specifically for attributes such as creaminess and smoothness. These sensorial attributes are crucial for consumer acceptance and preference (Fuhrmann et al., 2020; Liu, Stieger, van der Linden and van de Velde, 2015; McClements and Jafari, 2018; van Aken et al., 2011). Despite increasing research on plant proteins as emulsifiers, limited attention has been directed toward understanding how the mechanical stability of emulsions influences lubrication performance during oral processing.

The stability of oil droplets primarily depends on the interfacial layer of emulsifiers surrounding them, although other factors such as the viscosity of the continuous phase, temperature, pH, and ionic strength also play a role. Among these factors, the nature of the interfacial layer largely governs the degree of protection against coalescence. Small molecular weight emulsifiers tend to form a dynamic protective layer with relatively high mobility (Chung et al., 2018; McClements and Gumus, 2016; Tabaniag et al., 2023). Large molecular weight emulsifiers, such as proteins, tend to form a more solid-like protective layer at the oil-water interface (McClements and Jafari, 2018). Such an interfacial layer is able to withstand higher mechanical forces, and, therefore, often more efficient in preventing oil droplet coalescence (Ji et al., 2022a, Ji et al., 2022b). The viscoelastic properties of the protective interfacial layer depend on the conformation of the proteins and their in-plane protein-protein interactions at the interface. For instance, animal-based proteins, such as globular whey proteins (mostly β-lactoglobulin), can rearrange their 3D structure to some degree after migrating to the oil-water interface (Lam and Nickerson, 2013; Matsuyama et al., 2021; McClements and Gumus, 2016; Sagis and Yang, 2022). These globular, soluble animal-based proteins generally display strong in-plane protein-protein interactions at the oil-water interface, leading to self-assembly and thereby forming stiff viscoelastic interfacial films, which provide good mechanical stabilization against droplet deformation and coalescence under external forces (Ji et al., 2022). If proteins are in more aggregated form, they may provide less effective coverage of the interface and fewer interaction points, leading to weaker in-plane interactions and finally decreased stiffness of the oil-water interface. This higher level of aggregation is often seen for plant-based proteins. An example is pea proteins, which tend to have more complex protein conformations, due to their origin and extraction and drying steps, leading to protein aggregation and weaker interfacial films and lower oil droplet coalescence stability (Hinderink et al., 2020; Sagis and Yang, 2022; Zhang et al., 2023).

Incorporating protein-stabilized emulsions into food systems requires careful consideration of the resulting mouthfeel, as variations in mechanical properties of the oil-water interface can lead to differing degrees of droplet destabilization during oral processing, ultimately influencing consumer acceptance and preference. A key attribute, namely creaminess, is often lacking in plant-based foods but is known to correlate strongly with the lubrication properties of emulsions (Dickinson, 2018; Dresselhuis et al., 2008; Ji et al., 2023; Sarkar and Krop, 2019; Shewan et al., 2020; Stokes et al., 2013). The lubrication ability of foods relates to their capacity to lubricate in-mouth surfaces, such as the tongue and palate, preventing them from coming into direct contact during oral processing. A better understanding of the underlying mechanisms governing plant-based food lubrication could facilitate improvements in product development, thereby narrowing the sensory differences between plant-based and animal-based foods and ultimately enhancing consumer acceptance and satisfaction. In our previous study, we found that the lubricating capacity of dairy emulsions is strongly influenced by emulsifiers, which is attributed to variations in the mechanical stability of oil droplets under mechanical stress and leads to different lubrication mechanisms (Ji et al., 2022).

Unlike animal proteins, plant proteins exhibit a wide range of protein structural states, including their native structures (e.g., their monomeric, trimeric, or hexameric states) and aggregated forms resulting from processing steps, such as alkaline pH induced protein-phenol interactions, isoelectric point precipitation and heating (Yang et al., 2024). These differences can significantly affect the interfacial properties of emulsions as well as lubrication properties. While plant protein can exhibit promising lubrication compared to animal protein such as whey protein isolate (Zembyla et al., 2021), the aggregated forms could have drawbacks regarding lubrication, where they have been found to lead to high friction in emulsion systems. For instance, it was found that creams stabilized by pea isolate were shown to reduce friction more effectively than those formed with soy protein or faba protein isolate. Emulsions stabilized with heavily aggregated soy protein and faba bean protein demonstrated higher friction than emulsions stabilized with less aggregated pea protein (Ningtyas et al., 2021). In addition to the effects caused by different plant sources, various fractionation methods for plant proteins can also greatly impact their composition and functionality. Intensive fractionation processes could lead to protein denaturation and aggregation, which may decrease the lubricating capacity of protein-stabilized emulsion systems and result in undesired mouthfeel compared to those made with mildly refined pea protein (Zhou et al., 2025).

To date, while oil-water interfacial properties and lubrication behavior of plant protein-stabilized emulsions have been extensively studied, their correlation has been less explored, despite their close relation. A significant knowledge gap remains regarding how the oil-water interfacial properties in protein-stabilized (oil-in-water) emulsions affect their lubrication behavior, particularly in relation to droplet stability under mechanical stress. The tendency of emulsions to undergo coalescence under stress plays a critical role in the formation of oil films and the enhancement of lubrication, as previously reported by several studies (Ji et al., 2022; Nikolaou et al., 2024).

Our study aimed to elucidate further how the mechanical stability of the oil-water interface of emulsion droplets relates to their lubrication performance, by employing four types of proteins as models. Commonly used plant proteins, including soy protein isolate and two types of pea protein isolate extracted using different methods, were selected to prepare plant-based emulsions. Whey protein isolate served as a dairy-based reference. The lubrication behavior of the emulsions was investigated using tribological techniques. The mechanical properties of protein-stabilized oil-water interfacial films were assessed through advanced interfacial dilatational rheology, with the results further analyzed using general stress decomposition.

## 2 Materials and methods

### 2.1 Materials

Whey protein isolate powder (WPI, 90.5 % w/w purity, N × 6.38) was obtained from Davisco Foods International Inc. (Le Sueur, USA). Commercial pea protein isolate NUTRALYS s85F (PPIC, 78.7 % w/w purity, N × 5.7) was obtained from Roquette (Lestrem, France). Commercial soy protein isolate Unisol NRG IP Non-GMOW (SPI, 79.0 % w/w purity, N × 5.7) was obtained from Barentz International B.V. (Hoofddorp, the Netherlands). Lab-extracted pea protein isolate (PPIL, 87.3 % w/w purity, N × 5.7) was obtained using a previously developed method (Kornet et al., 2020). Medium Chain Triglycerides (MCT) oil was obtained from CREMER OLEO GmbH & Co. KG (Hamburg, Germany). All other chemicals were obtained from Merck (Darmstadt, Germany). All samples were prepared with ultra-pure Milli-Q water.

### 2.2 Sample preparation

#### 2.2.1 Preparation of WPI solutions

A 10 % (w/w) WPI stock solution was prepared by dissolving WPI powder in Milli-Q water and stirring overnight at 4 °C, followed by pH adjustment to 7.0. Afterwards, the solution was homogenized for three passes at 200 bar with a homogenizer (Delta Instrument, Lab homogenizer, The Netherlands) to break possible agglomerates. Subsequently, the solution was filtered through a membrane filter (cellulose acetate membrane filter, Whatman, GmbH Germany) with a pore size of 1.2 μm to remove any remaining clusters. At the end, the solution was diluted with Milli-Q water to obtain the final solutions that were stirred at room temperature (20 ± 0.1 °C) for 2 h. The final protein concentration was checked using Dumas (N × 6.38).

#### 2.2.2 Preparation of PPI and SPI solutions

PPIL (6 wt%), PPIC (8 wt%), and SPI (8 wt%) were prepared in 10 mM phosphate buffer (pH = 7.0, NaH_2_PO_4_·H_2_O and Na_2_HPO_4_) and stirred for at least 48 h at 4 °C, based on a previous method (Hinderink et al., 2019). The insoluble parts were removed by centrifugation at 16000×*g* for 30 min. The supernatant was centrifuged for a second time, and the protein concentration of the final supernatant was checked using Dumas (N × 5.7) (Flash EA 1112 Series Dumas, Interscience, The Netherlands).

#### 2.2.3 Preparation of oil-in-water emulsions

Oil-in-water stock emulsions (10 wt% MCT oil) were prepared by using four different proteins with a concentration of 1 wt% as emulsifiers: WPI, PPIL, PPIC, and SPI. MCT oil was slowly added into the aqueous protein phase, and the mixture was pre-homogenized with a rotor-stator homogenizer (Ultra Turrax T25, IKA Werke, Germany) at 13000 rpm for 3 min. Afterwards, the pre-emulsion was homogenized with a homogenizer (Delta Instrument, Lab homogenizer, the Netherlands) at 200 bar for 10 passes to obtain the final emulsion at room temperature (20 ± 0.1 °C).

### 2.3 Sample characterization

#### 2.3.1 Particle size distribution of emulsion droplets and proteins

The particle size distribution of the emulsion droplets was measured by static light scattering (MasterSizer2000, Malvern Instruments Ltd., Worcestershire, Malvern, UK). The refractive index for the dispersed phase (oil) and dispersant (water) was set to 1.45 and 1.33, respectively. The obscuration was 10 %. All the measurements were performed in triplicate using independent samples, and particle size distribution is reported as the volume-based average of triplicate measurements.

The particle size of the protein solutions was measured by dynamic light scattering using a Nano ZS (Zetasizer Ultra, Malvern Instruments Ltd., Worcestershire, Malvern, UK). The refractive index (RI) was set at 1.45 for protein. All the measurements were performed in triplicate using independent samples, and particle size distribution is reported as the volume-based average of triplicate measurements.

#### 2.3.2 Surface hydrophobicity

The hydrophobicity of protein dispersions (WPI, PPIL, PPIC, and SPI) was measured based on the method of Kato et al. (Kato and Nakai, 1980) with modifications. A series of protein concentrations (0.05, 0.1, 0.2, 0.5, 1, and 2 mg/mL) and a stock solution of the fluorescent marker 1-anilinonaphthalene-8-sulfonic acid (ANS, 8.0 mmol/L) were prepared. Each solution (5 mL) was mixed with 40 μL of ANS solution and incubated for 40 min. The fluorescence intensity (FI) was measured by a F-4600 fluorescence spectrophotometer (Hitachi, Japan) at wavelengths of 390 nm (excitation) and 470 nm (emission). The FI was plotted as a function of protein concentration, and the slope was calculated using a linear regression analysis to calculate the index of protein surface hydrophobicity (H_0_). All measurements were performed in triplicate using independent samples at room temperature (20 ± 0.1 °C).

#### 2.3.3 Oil-water interfacial properties

##### 2.3.3.1 Interfacial dilatational rheology

Interfacial characteristics at the oil-water interface were measured using an automated drop tensiometer (ADT, Teclis, France). A 30 mm^2^ area hanging water droplet was created at the tip of a G18-coated needle, which was immersed in a continuous MCT oil phase. The densities of the aqueous and oil phases were set at 0.998 and 0.951 kg/m^3^, respectively. Droplets containing MilliQ water or 1 % protein solution were created and monitored for 1 h. The droplet shape was analyzed and fitted with the Young-Laplace equation to obtain the interfacial tension values, using the software provided by the supplier.

After this adsorption period, interfacial dilatational rheology was performed through oscillatory dilatational deformations in either an amplitude or frequency sweep. The amplitude sweep was performed by increasing the deformation amplitude from 2.5 to 50 % area deformation at a frequency of 0.02 Hz. The frequency sweep was performed by increasing the frequency from 0.005 to 0.1 Hz at a deformation amplitude of 5 %. Each step consisted of five oscillation cycles, separated by a pause equal in duration to the previous cycle. All measurements were performed in triplicate using independent samples at 20 °C.

##### 2.3.3.2 Data analysis and general stress decomposition

The interfacial stress output was qualitatively analyzed using Lissajous plots of the interfacial stress (γ-γ_0_) against the deformation ((A-A_0_)/A_0_). The γ and A are the interfacial tension and area of the deformed interface, γ_0_ and A_0_ are the interfacial tension and area of the non-deformed interface. The middle three oscillations were used to create the plots. Quantitative analysis on the interfacial stress response obtained from amplitude sweeps was performed by a recently developed general stress decomposition (GSD) method (de Groot et al., 2024), in which the interfacial stress is decomposed into odd and even harmonics by Fourier Transformation, using an in-house developed MATLAB script (MATLAB, 2022b) to process the data. In the Fourier series, higher harmonics (up to 8) were included only when their relative contribution to the total harmonic intensity was at least 3 %. The generated harmonics were then used as input to create the separate contribution of the odd (equation (1)) and even harmonics (equation (2)), giving the interfacial pressure of the contribution of the odd ($\Pi {\left(t\right)}_{odd}$) and even ($\Pi {\left(t\right)}_{even}$) harmonics:

(1) $$\Pi {\left(t\right)}_{odd}=\tau_{1}+\tau_{2}=\sum_{k=0}^{\frac{n-1}{2}}b_{2k+1}^{\prime }\;\sin\left(\left(2k+1\right)\omega t\right)+\sum_{k=0}^{\frac{n-1}{2}}a_{2k+1}^{\prime }\;\cos\left(\left(2k+1\right)\omega t\right)$$

(2) $$\Pi {\left(t\right)}_{even}=\tau_{3}+\tau_{4}=\sum_{k=0}^{\frac{m}{2}}c_{2k}^{\prime }\;\sin\left(2k\omega t\right)+\sum_{k=0}^{\frac{m}{2}}d_{2k}^{\prime }\;\cos\left(2k\omega t\right)$$

Here, ω is the frequency of deformation, *t* is the time, and *n* and *m* are the highest included odd and even harmonic, respectively. The odd harmonics consist of *τ*_*1*_ and *τ*_*2*_, which are the elastic and viscous contributions to the total interfacial pressure as a result of stretching and compressing of the network structure formed by the protein, with Fourier coefficients *b'*_*2k+1*_ and *a'*_*2k+1*_, respectively. The even harmonics consist of *τ*_*3*_ and *τ*_*4*_, which are the viscous and elastic contribution to the interfacial pressure due to interfacial density changes (and hence are a result of the non-linearity of the interfacial pressure isotherm) with Fourier coefficients c*'*_*2k*_ and d*'*_*2k*_, respectively. The decomposed signals can be quantified using equations (3)–(6):

(3) $$E_{\tau 1L}=\frac{\sum_{k=0}^{\left(n-1\right)/2}b_{2k+1}^{\prime }{\left(-1\right)}^{k}}{\varepsilon_{0}}$$

(4) $$U_{d\tau 2}=\pi \;\varepsilon_{0}^{2}\;E_{1}^{\prime \prime }$$

(5) $$E_{\tau 4}=-\frac{\sum_{k=0}^{m/2}2d_{4k+2}^{\prime }}{\varepsilon_{0}}$$

(6) $$U_{d\tau 3}=2\varepsilon_{0}^{2}\;\sum_{k=1}^{m/2}\left(\frac{E_{2k\tau 3}*k}{k^{2}-1/4}\right)$$

Here, *E*_*τ1L*_ is the elastic modulus at maximum strain of the odd harmonics. *U*_*dτ2*_ describes the intracycle viscous dissipation of the odd harmonics. The even harmonics are quantified by the elastic modulus *E*_*τ4*_ and the dissipated energy *U*_*dτ3*_. Other parameters: $\varepsilon_{0}$ is the strain amplitude of the applied deformation ($\varepsilon =\varepsilon_{0}\;\sin\left(\omega t\right)$), *E″*_*1*_ is the loss modulus of the first harmonic, and *E*_*2kτ3*_ is the modulus of each harmonic contained in *τ*_*3*_. We refer to the original article and a recently published review for in-depth explanations about the parameters (de Groot et al., 2023; Risse et al., 2025).

#### 2.3.4 Viscosity measurement

The viscosity of the samples was measured using a stress-controlled rheometer (MCR 302, Anton Paar, Austria) with a double gap geometry (probe DG.26.7/Ti; cup DG 26.7/T200/Ti). A sample of 3.8 mL was pipetted into the cup and equilibrated at 20 °C for 5 min before the measurement started. The shear rate was increased in 5 min with logarithmic steps from 0.1 s^−1^–1000 s^−1^, and then decreased from 1000 s^−1^ to 0.1 s^−1^ in 5 min. All the measurements were performed in triplicate using independent samples, and the average value of the viscosity was determined.

#### 2.3.5 Tribology measurement

Tribological tests were conducted on a stress-controlled rheometer (MCR 302, Anton Paar, Austria) equipped with a tribology cell (T-PTD 200, BC 12.7, Anton Paar, Austria). The configuration followed a glass ball-on-three-pins design, consisting of a glass sphere (d = 12.7 mm) and three PDMS pins (d = 6 mm, roughness 0.2 μm ± 0.03). Experiments were performed at 20 °C under a normal force, F_N_, of 1 N. Samples (0.6 ml) were placed in the tribology cup, and the coefficient of friction (*μ*) was recorded as a function of sliding speed (0.01–470 mm/s). Each measurement comprised two cycles (four runs in total with increasing-decreasing-increasing-decreasing sliding speed, 5 min per run); the first run was discarded due to variability, and data from the third run was used for further analysis. All measurements were performed in triplicate with fresh samples. The mean friction coefficient (*μ*) was plotted against sliding speed (*v*_s_), and its decline in the mixed lubrication regime was quantified using a power-law model showing in equation (7):

(7) *μ ∼ v*_*s*_^*m*^

### 2.4 Statistical analysis

All measurements were tested in triplicates, and the results are expressed in the form of mean and standard deviation. Descriptive statistics (mean and standard deviation) were calculated for each sliding speed to illustrate trends and variability in lubrication behavior. Scatter plots were used to explore the relationship between sliding speed and friction coefficient. Since the dataset consists of continuous variables across a sliding speed range, ANOVA could not be applied on the entire dataset. Instead, one-way ANOVA was performed on friction coefficients at selected speeds (1, 10, 100, and 400 mm/s). Figures were made using OriginLab (Origin, 2018) and Python (Python 3.9.18) and statistical analysis were performed with Python (Python 3.9.18), MATLAB (MATLAB, 2022b), and SPSS (IBM SPSS Statistics27).

## 3 Results and discussion

### 3.1 Protein molecular properties: particle size and hydrophobicity

**Fig. 1:**
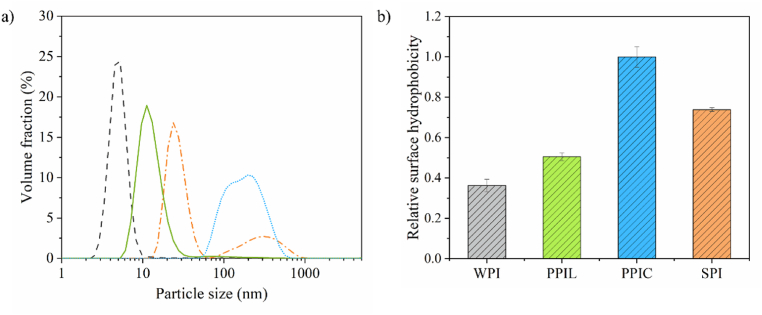
(a) Particle size distribution of a 1 % (w/w) soluble fraction of whey protein isolate (WPI, grey), lab-extracted pea protein isolate (PPIL, green), commercial pea protein isolate (PPIC, blue) and soy protein isolate (SPI, orange) in 20 mM PO_4_ buffer. Obtained using the zeta-sizer. For clarity, one representative graph is shown here based on three comparable triplicate measurements. (b) Surface hydrophobicity of the earlier mentioned samples. The mean and standard deviation are obtained from triplicate measurements.

The emulsions created in this work were compared based on soluble protein content. While whey protein isolate (WPI) was nearly fully soluble (93 %), the soy protein isolate (SPI), commercial pea protein isolate (PPIC) and lab-extracted pea protein isolate (PPIL) showed lower dispersibility of 60 %, 31 %, and 38 %, respectively. The lower dispersibility can be explained by analyzing molecular properties such as particle size and surface hydrophobicity of soluble fraction of the protein solutions (Fig. 1).

WPI and PPIL show a monodisperse and narrow peak with particle size at the peak measuring 5.3 and 11.2 nm, respectively (Fig. 1a). SPI shows a bimodal distribution with a narrow peak at 23.9 nm, and a broad secondary peak between 80 and 893 nm. The PPIC showed a wide size range between 60 and 660 nm, with a peak of 197.6 nm.

WPI has the smallest particle size, which corresponds to the size of β-lactoglobulin in a dimeric state (Aymard et al., 1996; Yang, Thielen, Berton-Carabin, van der Linden and Sagis, 2020). The size of PPIL corresponds to the size of the native globulin proteins vicilin and legumin (Kornet et al., 2020). The substantially larger size of PPIC is the result of protein aggregate formation. The difference between PPIL and PPIC, although both are extracted from peas, can be attributed to the production method. While both were extracted using an alkaline extraction - acid precipitation method, PPIL was non-heated and freeze-dried, where there is little exposure to elevated temperatures. The commercial PPIC was most likely heated to ensure microbial stability and was also spray-dried. Heating induces the disruption of hydrogen bonds, leading to the unfolding of the protein structure, and finally inducing the aggregation of exposed hydrophobic sites (Betz et al., 2012). This would explain the larger aggregates of the commercial PPIC, compared to the more dispersible PPIL (Yang et al., 2022). The commercially obtained SPI (most likely also exposed to elevated temperatures) shows an intermediate behavior, where a peak is observed near the size of globulins, and another peak with a larger size reflects the presence of aggregates. The SPI might be less heavily aggregated than the PPIC, also presenting more dispersible proteins.

The protein surface hydrophobicity of the soluble fraction, as shown in Fig. 1b, was measured as a relative value, normalized to the sample with the highest surface hydrophobicity (set as 1.0). The relative surface hydrophobicity values of protein molecules were 0.36, 0.50, 1.0, and 0.73 for WPI, PPIL, PPIC, and SPI, respectively. WPI and PPIL have the lowest surface hydrophobicity values, reflecting a more native globular structure. This is consistent with their smaller size (Fig. 1a). SPI and PPIC show significantly higher values, about 2–3 times higher than WPI and PPIL, confirming our hypothesis that heat exposure during the commercial process leads to exposure of the hydrophobic sites and the accompanying aggregation (Fig. 1a).

### 3.2 Oil-water interfacial properties: rheological deformations and non-linear behavior analysis

The oil-water interface-stabilizing properties of the four protein samples were studied using drop tensiometer. First, the oil-water interface adsorption behavior of the proteins was evaluated, and the results are shown in Fig. S1 in the supplementary data. All protein samples showed high interfacial activity with interfacial pressure values at 1 s ranging from 4.8 to 9.0 mN/m. After 1 h of adsorption time, the protein-stabilized oil-water interfacial films were subjected to large amplitude oscillatory dilatational deformations.

#### 3.2.1 Dilatational oscillatory deformations - frequency and amplitude sweeps

**Fig. 2:**
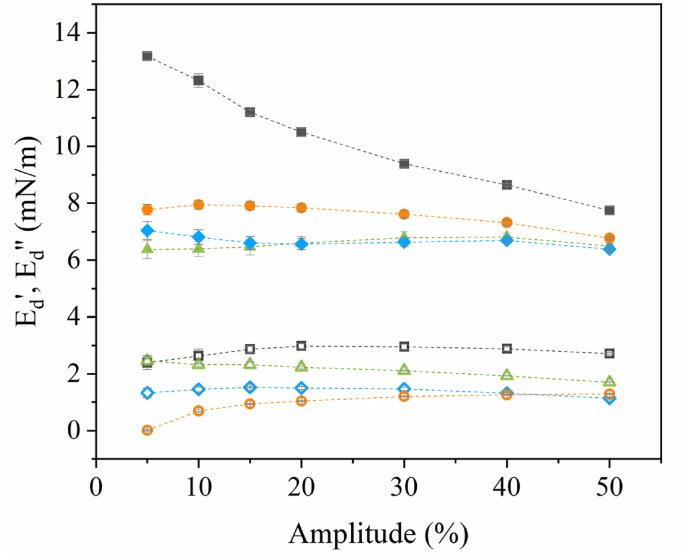
The dilatational elastic (*E*_*d*_*'*) and viscous (*E*_*d*_*''*) moduli as a function of deformation amplitude of MCT oil-water interfacial films, stabilized by 0.1 % (w/w) soluble fraction of whey protein isolate (WPI, *E*_*d*_*':* grey square, *E*_*d*_*'':* open grey square), lab-extracted pea protein isolate (PPIL, *E*_*d*_*':* green triangle, *E*_*d*_*'':* open green triangle), commercial pea protein isolate (PPIC, *E*_*d*_*':* blue diamond, *E*_*d*_*'':* open blue diamond) and soy protein isolate (SPI, *E*_*d*_*':* orange circle, *E*_*d*_*'':* open orange circle) in 20 mM PO_4_ buffer. The n-value of WPI, PPIL, PPIC and SPI stabilized MCT oil-water interfacial films is shown in the insert. The mean and standard deviation are obtained from triplicate measurements.

The mechanical properties of protein-stabilized interfacial films were further studied by performing frequency and amplitude sweeps (Fig. 2). Frequency sweeps of the interfacial films were performed to study the frequency dependency of the moduli. When plotting the *E*_*d*_*'* over frequency (*ω*, in Hz) (Fig. S2, supplementary data), we observe a power-law behavior as *E*_*d*_*'* ∼ *ω*^*n*^. The exponent *n*, or n-value, quantifies the degree of frequency dependency and is directly related to the underlying interfacial dynamics, and has been described by the classical Lucassen-van den Tempel model (Lucassen & Van Den Tempel, 1972). According to this model, the frequency dependence of the dilatational modulus is controlled by the balance between two processes: 1. elastic restoring forces generated by the interfacial layer itself, and 2. exchange of surface active species between interface and bulk. When the interface is periodically deformed, changes in area induce concentration changes that are partially relaxed by diffusion and mass transport. If this relaxation is dominant, the n-value will be 0.5, indicating that the interfacial elasticity is mainly governed by mass exchange between the bulk and the interface during deformation. This behavior is characteristic for interfaces stabilized by small molecular surfactants. Oppositely, when the interfacial layer behaves as a cohesive network, exchange processes are too slow to follow the deformation, resulting in much lower n-values, as earlier observed for proteins due to their ability to form cohesive interfacial networks (Kornet, Yang, Venema, van der Linden and Sagis, 2022; Yang et al., 2020). The power-law exponent n provides direct physical insight into whether the interfacial response is governed by diffusion-controlled mass exchange or by in-plane network elasticity.

In our study, the WPI-stabilized interface had the lowest n-value of 0.13, followed by a value of 0.19 for PPIL, and these values suggest strong in-plane interactions between the adsorbed proteins. The SPI and PPIC showed higher n-values of 0.34 and 0.35, suggesting that the exchange dynamics between the bulk and interface contribute more strongly to the elasticity of these films.

Amplitude sweeps were performed to study the rheological behavior at increasing deformation amplitudes, and the results are shown in Fig. 2. All protein-stabilized oil-water interfacial films showed significant values for both dilatational elastic (*E*_*d*_*'*) and viscous (*E*_*d*_*''*) moduli, suggesting the formation of viscoelastic layers for all proteins. WPI had the highest *E*_*d*_*'* with values decreasing from 13.2 to 7.7 mN/m upon increasing the deformation amplitude from 5 to 50 %. Interfacial films stabilized by the other proteins showed substantially lower *E*_*d*_*'*, with values ranging from 6.8 to 7.8 mN/m for SPI, and 6.4–7.0 mN/m for PPIL and PPIC. WPI showed a strong decrease in moduli at higher deformations, suggesting disruption of an interfacial microstructure. This interfacial microstructure, which refers to the organization and connectivity of proteins within the interfacial layer, including clustering, packing and network development, could only be formed due to strong in-plane interactions between proteins, which is also reflected in the low n-value of 0.13. Such behavior for WPI has been shown before for both oil-water and air-water interfaces (Yang et al., 2023; Zhou et al., 2021; Zhou et al., 2020). The lower *E*_*d*_*’*-values for SPI and PPIC suggest substantially weaker interfaces. This in combination with their high n-values, close to 0.5, would suggest the formation of a weak and highly mobile interface, where proteins might desorb from interface into the bulk upon compression and adsorb again upon expansion. Interestingly, PPIL had a similarly low moduli as the PPIC, but showed a nearly twofold lower n-value. We will further discuss this difference in the next sections, where we will dive deeper into the non-linear contributions.

#### 3.2.2 Qualitative analysis of non-linear behavior - Lissajous plots

**Fig. 3:**
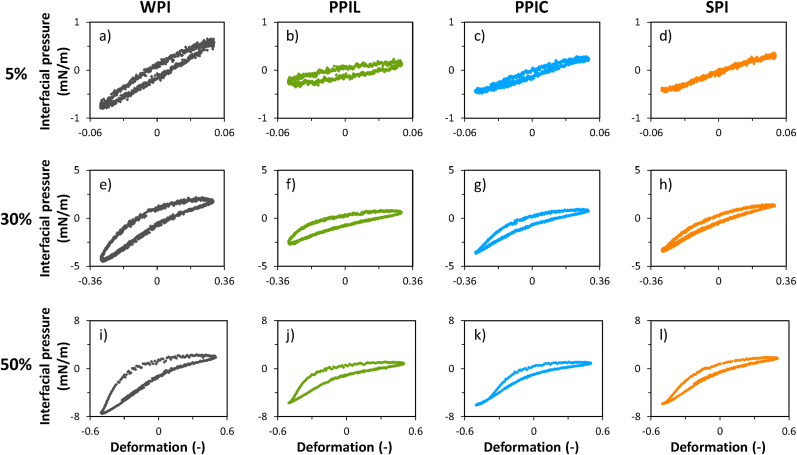
Lissajous plots of interfacial pressure as a function of applied deformation (5, 30 and 50 %), which was obtained from amplitude sweeps of MCT oil-water interfacial films stabilized by 0.1 % (w/w) soluble fraction of whey protein isolate (WPI), soy protein isolate (SPI), lab-extracted pea protein isolate (PPIL) and commercial pea protein isolate (PPIC) in 20 mM PO_4_ buffer. For clarity, one representative graph is shown here taken from three comparable triplicate measurements.

The large dilatational deformations in the amplitude sweeps could disrupt interfacial microstructures, thereby generating non-linear viscoelastic (NLVE) behavior. This generation of non-linearities will lead to higher-order harmonics in the Fourier spectrum of the interfacial stress response. These higher-order harmonics are not taken into account when calculating the (first harmonic-based) moduli *E*_*d*_*'* and *E*_*d*_*''*, thus neglecting the non-linear viscoelastic contributions, while these contributions might possess crucial insights into the mechanical properties of interfacial films (Sagis et al., 2014). First, we will qualitatively analyze the raw data by constructing Lissajous plots (Risse et al., 2025), where the interfacial stress is plotted as a function of deformation of the interfacial area. Lissajous plots of WPI, PPIL, PPIC and SPI are shown in Fig. 3.

Before discussing the results, we will provide a few basic interpretations. 1) In the plots the interfacial pressure changes in a clockwise direction, where the upper part of the plot is the extension cycle, and the opposite (compression) occurs at the bottom part of the plot. 2) An important characteristic is the width of the plots, showing the type of rheological response. A straight line (closed plot) reveals a fully elastic response; a circle implies a fully viscous response, while an intermediate linear viscoelastic response is reflected as an ellipse. 3) Another parameter is the angle of plots, which is related to the layer stiffness, where a steeper plot reflects a stiffer interface. 4) Finally, asymmetries between the extension and compression cycle can be present, which are the result of the non-linear contributions, and will provide critical insights on molecular interactions among adsorbed proteins.

At 5 % deformation, the WPI-stabilized interface (Fig. 3a) showed narrow symmetric ellipses, revealing a predominantly elastic response. At higher deformations, the plots of WPI started to show asymmetries, which were most pronounced for the WPI-stabilized interfacial film at 50 % deformation (Fig. 3i). In the extension cycle, we observe a steep increase of interfacial pressure for WPI, followed by a rapid flattening of the curve. This is known as intra-cycle strain softening in extension, which results from gradual disruption (and softening) of the microstructure upon substantial expansion (or stretching) of the interfacial area. In the compression cycle, the opposite can be seen, here, the interfacial pressure rapidly decreases to −8 mN/m, a phenomenon known as strain hardening in compression. This could be attributed to the extensive compression of proteins, without expulsion into the bulk, leading to high protein densities, and, thus, a more negative interfacial pressure. This behavior is typically seen for stiff solid-like interfacial films, where the adsorbed proteins have strong in-plane interactions, as shown for other whey protein stabilized oil-water interfaces (Hinderink, Sagis, Schroën and Berton-Carabin, 2021a; Zhou et al., 2020).

The PPIL-, PPIC- and SPI-stabilized interfaces also showed narrow symmetric ellipses at 5 % deformation (Fig. 3b–d). At 50 % deformation, the Lissajous plots of these three proteins show comparable plots (Fig. 3j–l), but show differences with that of WPI. The main difference is a lesser extent of strain hardening in compression, which could indicate that interfaces stabilized by PPIL, PPIC and SPI could not be compressed till such high protein densities as for WPI. This suggests that more material desorbs again into the bulk, which aligns with the higher frequency dependency found for PPIL, PPIC and SPI compared to that of WPI (Fig. 2). Although the results under compression are similar for PPIL, PPIC and SPI (Fig. 3j–l), small differences in the interfacial pressure during expansion are observed for the three proteins. However, it is difficult to draw qualitative conclusions from these minor differences. To gain more insights, we quantified these plots further to allow for a better comparison, which will be further discussed in the next section.

#### 3.2.3 Quantitative analysis of non-linear behavior - general stress decomposition

**Fig. 4:**
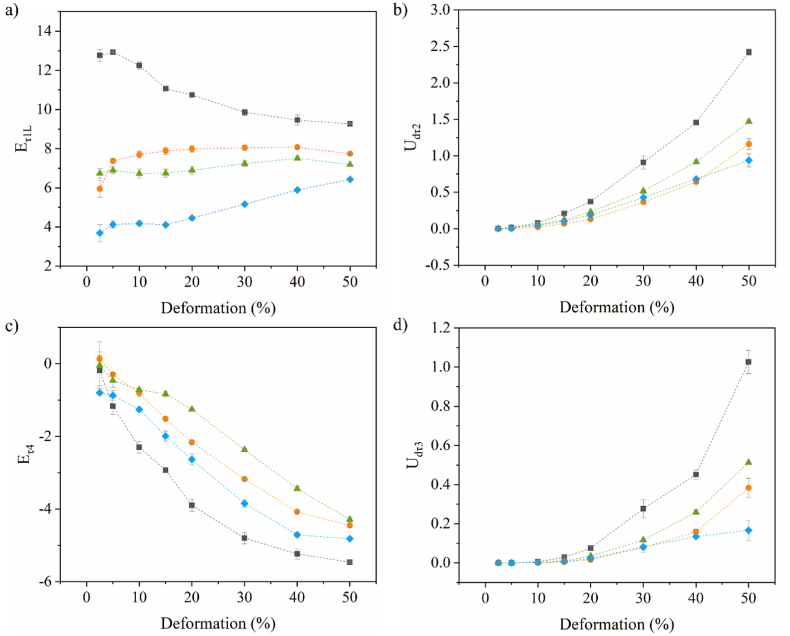
(a) E_τ1L_, (b) U_dτ2_, (c) E_τ4_ and (d) U_dτ3_ as a function of deformation amplitude of MCT oil-water interfacial films, stabilized by 0.1 % (w/w) soluble fraction of whey protein isolate (WPI, grey square), lab-extracted pea protein isolate (PPIL, blue diamond), commercially-available pea protein isolate (PPIC, green triangle) and soy protein isolate (SPI, orange circle) in 20 mM PO_4_ buffer. The mean and standard deviation are obtained from triplicate measurements.

The asymmetries shown in the Lissajous plots in Fig. 3 are unique for dilatational rheology. In dilatation, the asymmetry is introduced due to changes in interfacial density of the adsorbed proteins, which increases in compression and decreases in expansion. As a result, a Fourier transform of the stress response will result in a spectrum with odd and even harmonics. Using a general stress decomposition, the odd and even harmonics can be split, where the odd harmonics show the contribution of network interactions to the stress response, and the even harmonics represent the contribution from interfacial density changes. The odd harmonics give an elastic and viscous contribution τ_1_ and τ_2_, respectively, and the even harmonics yield an energy-storage and dissipating component, which are τ_4_ and τ_3_, respectively. The Lissajous plots created from the four signals were then quantified based on equations (3)–(6), and the results are shown in Fig. 4. We refer to previous work for details about the general stress decomposition in dilatational interfacial rheology (de Groot et al., 2023). This analysis reveals the underlying elastic and viscous stress contributions during nonlinear deformations, providing mechanistic insights into interfacial film breakdown that is not accessible from conventional (first-harmonic-based) amplitude or frequency sweeps.

First, we start with the contributions of the odd harmonics of a WPI-stabilized interface. Fig. 4a shows the E_τ1L_, which is the modulus which quantifies the network interactions. WPI shows the highest initial values followed by a steep decrease, suggesting the strongest network interactions, and structure breakdown at larger deformations. The disruption of the WPI network is also evident from the increase of dissipated energy U_dτ2_ at higher deformations (Fig. 4b). In the even harmonics, WPI showed the (absolute) highest E_τ4_ modulus (Fig. 4c), which is a quantification of the elastic component of the interfacial density contribution. This component also has a viscous component, which is quantified as U_dτ3_ (Fig. 4d), where WPI also showed the highest values. Such observations suggest strong density contributions of whey proteins to the interfacial pressure response, and hence a steep interfacial pressure isotherm in the range of applied deformations. Whey proteins are able to form strong interfacial networks, where the adsorbed proteins remain on the interface upon large (compression and extension) deformations, and such behavior would only be possible if strong in-plane interactions are present between the adsorbed proteins (Dickinson et al., 1990).

For PPIC and SPI, we observe lower E_τ1L_ moduli (Fig. 4a) and dissipation U_dτ2_ (Fig. 4b) than for WPI, thereby indicating the formation of a weaker network and hence weaker interactions between the adsorbed proteins. The lower contribution by the density-related components E_τ4_ (Fig. 4c) and U_dτ3_ (Fig. 4d) than for WPI is consistent with this. In addition, there could be more material exchange between the bulk and interface during large deformations, partially compensating the changes in interfacial density, which is in line with the higher frequency dependency found during the frequency sweeps (Fig. 2). These findings point towards much weaker interactions between adsorbed SPI and PPIC proteins, which will form an interface with lower overall stiffness. This could be attributed to the heavily aggregated protein state, as shown by larger sizes in the particle size distribution (Fig. 1a). Such large aggregates cover the interfacial area less effectively, providing less in-plane interaction between adsorbed proteins, leading to weaker interfaces (Yang et al., 2022). The observed difference would lead to differences in the deformability of the oil droplets, where the WPI stabilized ones are more likely to behave as harder spheres whereas those stabilized by plant proteins are softer.

PPIL proteins showed the (absolute) lowest values for E_τ1L_ (Fig. 4a) and E_τ4_ (Fig. 4c), but dissipation values in U_dτ2_ (Fig. 4b) and U_dτ3_ (Fig. 4d) were between those of WPI and SPI/PPIC. While the elastic contributions of network interactions and interfacial density are extremely low, relatively high dissipation can be observed for both contributions. The PPIL proteins at the interface seem to have very little in-plane interaction, but proteins are unlikely to be pushed into the bulk upon compression, as we obtained a relatively low n-value in the frequency sweep. The high dissipation could point towards a high deformability of the interface at high deformations. This may be related to the higher dispersibility of the PPIL. The larger number of more smaller and native proteins present in the solution (Fig. 1a) could contribute to the formation of a more deformable film. But these PPIL proteins are less able to give strong in-plane interactions compared to WPI. A major reason why WPI is able to form such stiff interfacial films is related to its flexibility, allowing reorganisation of interactive sites toward each other (Hinderink et al., 2020). PPIL mostly consists of globulins, which were previously described to be more rigid proteins, thus unable to form these stronger interactions (Hinderink, Berton-Carabin et al., 2021b). We want to highlight that the two works on Hinderink et al. used the same WPI as in this work, and the PPIL in both our works were extracted using similar methods. This could result in (PPIL) proteins that can interact to a certain extent at the oil-water interface that are more deformable.

The more quantitative analysis points out that the three plant proteins do show different behavior, mostly related to the desorption ability. In the next section, we will discuss the lubrication properties of emulsions stabilized by these proteins.

### 3.3 Emulsion properties: droplet size and viscosity

Emulsions were stabilized by the four studied protein isolates using the dispersible protein fraction. To investigate the effect of protein and interfacial properties, we aimed to keep oil droplet size and viscosity similar, as droplet size and viscosity have been shown to influence lubrication.

**Fig. 5:**
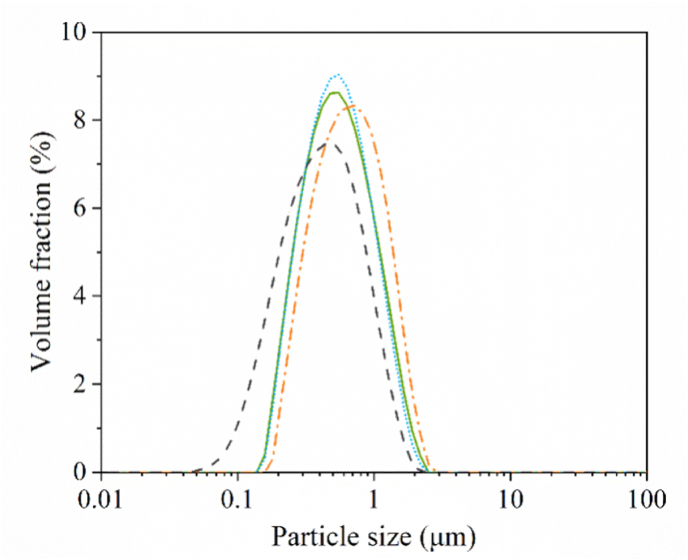
Volume-based droplet size distribution of 5 % oil content (w/w) emulsions stabilized with 1 % (w/w) soluble protein from whey protein isolate (WPI, dashed grey line), lab-extracted pea protein isolate (PPIL, solid green line), commercially-available pea protein isolate (PPIC, short-dotted blue line) and soy protein isolate (SPI, dash-dotted orange line) in 20 mM PO_4_ buffer. For clarity, one representative graph is shown taken from three comparable triplicate measurements.

The volume-based droplet size distribution of emulsions stabilized by WPI, PPIL, PPIC and SPI, is shown in Fig. 5. All four emulsions showed comparable droplet size distributions with a peak size between 0.5 and 0.7 μm. In addition, the viscosity of the four protein-stabilized emulsions (5 % oil content) was comparable with values between 1.3 and 1.5 mPa s at a shear rate of 1000 s^−1^ (data not shown). This shear rate was chosen for viscosity comparison because it reflects the high local shear and near thin-film conditions of tribological contacts (Fig. S3, supplementary data). Since the particle sizes and viscosity are comparable across all samples, differences in lubrication behavior are thus expected to originate from variations other than particle size and viscosity.

### 3.4 Tribological behavior - Effect of different source of protein as emulsifier

In general, emulsions can contribute to lubrication through distinct mechanisms, including the plate-out mechanism (film lubrication) and the rolling/sliding mechanism (particle-based lubrication). The plate-out mechanism in emulsions involves the coalescence of oil droplets, leading to the formation of oil patches or a continuous oil film on the contact surfaces (Ji et al., 2022; Liu, Tian, Stieger, van der Linden and van de Velde, 2016). Particle-based lubrication occurs when emulsion droplets behave as particles that are able to roll/slide between the two solid surfaces in relative motion (e.g., glass against a PDMS surface), thereby reducing friction between the two solid surfaces by acting as physical separators (Kew et al., 2023; Liu et al., 2016; Rudge, van de Sande, Dijksman and Scholten, 2020; Torres et al., 2018). Depending on the applied pressure and the mechanical properties of the oil droplets, they may deform to varying degrees but still function as physical spacers between the surfaces, reducing friction through rolling, sliding, or a combination of both. In contrast, oil droplets with lower mechanical stability may rupture and coalesce under mechanical stress. When droplets coalesce to form oil patches or a continuous oil layer on the interacting solid surfaces, this layer reduces direct contact between the surfaces and consequently decreases the friction coefficients. This phenomenon is known as the plate-out mechanism.

**Fig. 6:**
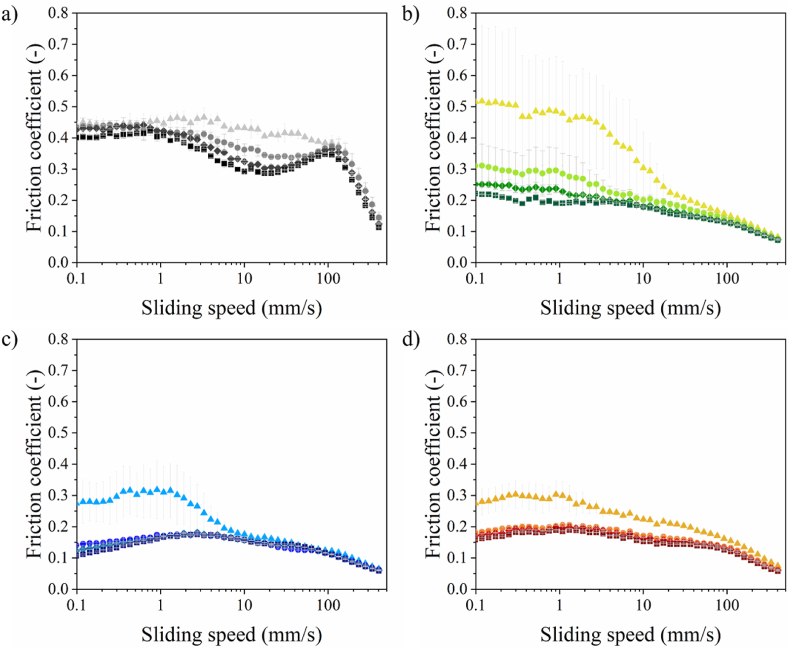
Friction coefficient as a function of sliding speed of a) WPI-stabilized, b) PPIL-stabilized, c) PPIC-stabilized, and d) SPI stabilized emulsions at an oil content of 2.5 (triangle), 5 (circle), 7.5 (diamond), and 10 % (square). Darkers color indicate higher emulsion concentrations. The mean and standard deviation were calculated from three independent measurements.

As shown in Fig. 6, the emulsions stabilized by different proteins exhibited different lubrication profiles and varying degrees of concentration dependency. Since the emulsions are homogeneous systems and the droplets sizes were similar, a higher oil content corresponds to a greater number of droplets. To be more specific, Fig. 6a shows that the friction coefficients of WPI-stabilized emulsion (WPI-E) exhibited a clear concentration dependency at the sliding speed range of 1–100 mm/s, where a higher oil content (i.e., a greater number of droplets) was associated with lower friction coefficients. We observed that WPI-E with 2.5 % oil demonstrated a distinct transition from the boundary regime, where the friction coefficient is independent of sliding speed, to the mixed regime, where the friction coefficient decreases with increasing sliding speed. For samples containing 5–10 % oil, the friction coefficient exhibited a non-monotonic response to increasing sliding speed. Initially, friction remained constant, indicating a short boundary regime. This was followed by a decrease, then a transient increase, and a subsequent decline, forming a characteristic “hump” in the lubrication profile (10–100 mm/s). Notably, the steepness of the “hump” increased with a higher concentration of oil droplets. This phenomenon is likely caused by the accumulation and jamming of emulsion droplets, leading to an increase in friction coefficient with a specific speed range. Such behavior seems specific of particulate systems, as this has been observed for other emulsions and particle suspensions (Ji et al., 2024; Ji et al., 2022). This behavior of WPI-E aligns with findings from our previous study, in which we demonstrated that the emulsion droplets stabilized by WPI stayed intact and served as physical separators between the two contacting surfaces without undergoing destabilization (Ji et al., 2022). It is worth mentioning that these separators may deviate from a spherical shape under pressure, providing lubrication through a rolling or sliding action, depending on the degree of deformation.

The hump observed in the frictional curve of WPI-stabilized emulsion was not present in the emulsions stabilized by PPIL (Fig. 6b). However, PPIL-stabilized emulsions (PPIL-E) also exhibited a clear concentration dependency, with lubrication performance improving greatly as the oil droplets concentration increased. The emulsion containing 10 % oil showed an initial friction coefficient of approximately 0.22, representing a 2.4-fold reduction compared to the 2.5 % oil samples (μ ∼ 0.52). The improved lubrication is likely attributed to the increased number density of emulsion droplets, meaning more droplets entered the gap between contact solid surfaces and contribute to surface separation. This suggests a particle-based mechanism in which droplets lubricate via rolling and/or sliding, and the lubrication behavior shows concentration dependence. Notably, the friction coefficients of the 2.5 % PPIC-E and SPI-E were lower than those of the 2.5 % WPI-E and PPIL-E. For emulsions such as PPIC-E and SPI-E, increasing the oil concentration from 2.5 % to 5 % resulted in a slight friction reduction. At concentrations above 5 %, the lubrication curves overlapped, indicating that further increases in oil content did not contribute to additional friction reduction. This phenomenon for PPIC-E and SPI-E is likely attributed to droplet coalescence. Coalesced oil droplets can form discrete oil patches on the interacting surfaces, leading to a reduction in friction. Once the surfaces became fully covered by coalesced oil droplets (at 5 % oil content in these cases), further increases in the oil content did not result in additional friction reduction. This behavior aligns with the plate-out lubrication mechanism, where a continuous oil film reaches a saturation point beyond which excess oil no longer contributes to further friction reduction, consistent with previous studies (Ji et al., 2022). Additionally, PPIC-E and SPI-E show very similar lubrication behavior, even though large aggregates are present in PPIC (Fig. 1). Although PPIC aggregates are relatively large compared to other proteins, they remain smaller than the emulsion droplets, suggesting that the droplets dominate the lubrication behavior.

As discussed in the previous sections regarding oil-water interfacial properties, different proteins behave differently at the oil-water interface, forming distinct interfacial protein layers that determine the capability of oil droplets to stand mechanical stress. Droplets that can resist high mechanical stress remain intact under the high-shear conditions of friction and thus maintain their particle integrity, whereas droplets that cannot withstand high mechanical stress tend to rupture (i.e., low mechanical stability), resulting in lubrication behavior different from that of particle-based lubrication. WPI can form a stiffer, more solid-like interface that can potentially withstand higher mechanical forces. Although PPIL did not form such a strong interface, it showed high deformability under large deformations, which also helped limit droplet coalescence. As a result, the droplets did not coalesce under high mechanical stress, allowing them to provide lubrication via particle-based lubrication, where the dispersed droplets facilitated smooth movement between the surfaces through rolling or sliding. In contrast, oil-water interfaces stabilized by PPIC or SPI lacked strong in-plane interactions and were unable to form highly deformable films, resulting in weaker layers with lower overall stiffness. These oil droplets were more prone to rupture under mechanical stress, leading to droplet coalescence. The coalesced droplets subsequently formed oil patches that filled asperities on the contacting surfaces, which were hypothesized to provide oil-film lubrication.

### 3.5 Correlation heatmap of physical properties

**Fig. 7:**
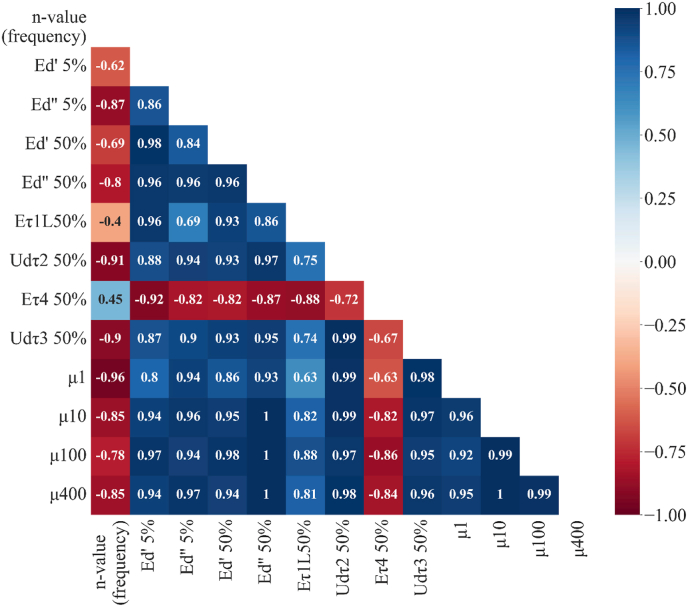
Correlation heatmap of lubrication properties (friction coefficient) and interfacial properties (n-value, *Ed' and Ed''* at 5 and 50 %; E_τ1M_, U_dτ2_, E_τ4_ and U_dτ3_ at 50 %) for emulsions stabilized by different sources of proteins. Lubrication parameters: friction coefficients at sliding speed of 1 mm/s (*μ*_*1*_), 10 mm/s (*μ*_*10*_), 100 mm/s (*μ*_*100*_), and 400 mm/s (*μ*_*400*_). Red indicates negative correlation and blue indicates positive correlation.

To examine further which oil-water interfacial properties are mostly responsible for the lubrication behavior, a correlation analysis was performed between the different interfacial properties and the friction coefficients at representative sliding speeds. As shown in Fig. 7, the n-value, representing the characteristics of the oil-water interface, is strongly and negatively correlated with the friction coefficients, particularly the friction coefficient at lower sliding speeds of 1 mm/s (correlation coefficient: −0.96), and relatively less at high speeds (10, 100, 400 mm/s). These findings support our hypothesis that a highly mobile interface (a high n-value) is associated with a lower friction coefficient. We propose that this relationship is related to the mechanical instability of emulsion droplets under stress, which facilitates emulsion droplet coalescence and the formation of a lubricating oil film. This suggests that analyzing the frequency-dependent behavior of the interfacial film may provide insights for screening emulsifiers with respect to their lubrication performance.

Additionally, the elastic dilatational moduli (*Ed'* and *Ed''*) at deformations between 5 and 50 % (only 5 % and 50 % shown in the map) are also highly correlated with the friction coefficients, especially at high sliding speeds above 10 mm/s, confirming that the high friction coefficients were associated with the observed stiffer oil-water interfaces. The same correlation was observed for U_dτ2_ and U_dτ3_, both of which exhibited a strong positive correlation with the friction coefficients, with correlation coefficient of 0.95 or higher. In addition, it was observed that a higher density contribution of protein at the oil-water interface contributes to low lubricating capacity. This is strengthened by a negative correlation between E_τ4_ and friction coefficient (i.e., high absolute E_τ4_ modulus corresponds to high friction coefficients). These results indicate that strong interfaces with high resistance against interfacial density change contribute less to lubrication, which is expected based on the findings in this work. It is important to highlight that, using four representative protein types with distinct interfacial properties, this study aimed to demonstrate that interfacial properties reflect dynamic structural changes in emulsions, which can be linked to their lubrication performance.

## 4 Conclusion

This work investigated how the protein layer at the oil-water interface of emulsion droplets relates to the emulsion's lubrication properties. Four commonly used proteins were evaluated: whey protein isolate (WPI), lab-obtained pea protein isolate (PPIL), commercially-obtained pea protein isolate (PPIC) or soy protein isolate (SPI). WPI formed stiff interfacial layers with low frequency dependency, indicating high mechanical stability of the oil droplets. Although PPIL formed a weaker oil-water interfacial layer, it was highly deformable, providing sufficient resistance for droplet coalescence. The lubrication behavior of emulsions stabilized by WPI or PPIL showed concentration dependence, with higher concentrations correspond to lower friction and thus higher lubrication capacity. We hypothesize that this emulsion lubrication behavior was governed by the presence of intact (stable) droplets, providing lubrication via rolling or sliding actions, depending on their degree of deformation under the applied load (particle-lubrication). In contrast, PPIC and SPI formed comparatively weak oil-water interfaces with high frequency dependence, due to weaker interactions between adsorbed proteins. Consequently, the droplets stabilized by PPIC or SPI showed low mechanical stability under stress, leading to droplet coalescence and resulting in lubrication via a plate-out mechanism.

By further exploring the correlation between the lubricating capacity and oil-water interfacial properties, we found that the lubrication properties showed high correlations with the n-value (frequency dependency), elastic dilatational moduli (*Ed'* and *Ed''*), viscous dissipation of the odd (U_dτ2_) and even (U_dτ3_) harmonics, as well as the elastic modulus of the even harmonics (Eτ_4_). These findings reveal valuable insights into the role of oil-water interfacial characteristics of oil droplets in governing lubrication behavior. Thus, these parameters can be used to evaluate the functionality of emulsifiers with respect to their contribution to lubrication behavior, which may offer opportunities for the food industry to improve mouthfeel characteristics in plant-based products formulations through more effectively targeted emulsifier selection and oil-water interface design. Emulsion droplets with weaker oil-water interfaces (PPIC and SPI) tend to generate lower friction coefficients than emulsions with stable droplets (WPI and PPIL), thereby enhancing lubrication and potentially facilitating fat-related mouthfeel attributes such as creaminess and smoothness in plant-based foods. However, overall system stability must be considered in food system applications. Thus, an appropriate balance between lubrication (associated with mouthfeel) and droplet stability is essential to achieve both desirable sensory attributes and stable food system.

## CRediT author statement

Lei Ji: Conceptualization, Investigation, Data curation, Methodology, Visualization, Writing- Original draft preparation. Leonard Sagis: Methodology, Validation, Writing - Review & Editing, Supervision. Elke Scholten: Methodology, Validation, Writing - Review & Editing, Supervision, Funding acquisition. Jack Yang: Conceptualization, Investigation, Data curation, Methodology, Visualization, Writing- Original draft preparation.

## Declaration of competing interest

The authors declared that no competing interests exist.
