# Supplementary material for: Lubrication by plant-based emulsions: Linking oil-water protein-stabilized interfacial mechanical properties to oil droplet lubrication properties
Source: Curr Res Food Sci. 2025 Dec 6;12:101270. doi: 10.1016/j.crfs.2025.101270 (PMC12753246; doi:10.1016/j.crfs.2025.101270)
Supplement: Multimedia component 1 [file mmc1.docx]

**Supplementary**





**Figure S1.** Surface pressure isotherms of a 0.1% (w/w) soluble fraction of whey protein isolate (WPI, grey square), lab-extracted pea protein isolate (PPIL, green triangle), commercial-available pea protein isolate (PPIC, blue diamond) and soy protein isolate (SPI, orange circle). The WPI was prepared in Milli-Q water, pH 7.0, while the PPIL, PPIC and SPI were prepared in 10 mM PO_4_ buffer. The total adsorption time was 3,600 s. For clarity, one representative graph was shown here from three comparable independent measurements.





**Figure S2.** Ed' as a function of frequency for whey protein isolate (WPI, grey square), lab-extracted pea protein isolate (PPIL, green triangle), commercial-available pea protein isolate (PPIC, blue diamond) and soy protein isolate (SPI, orange circle) .


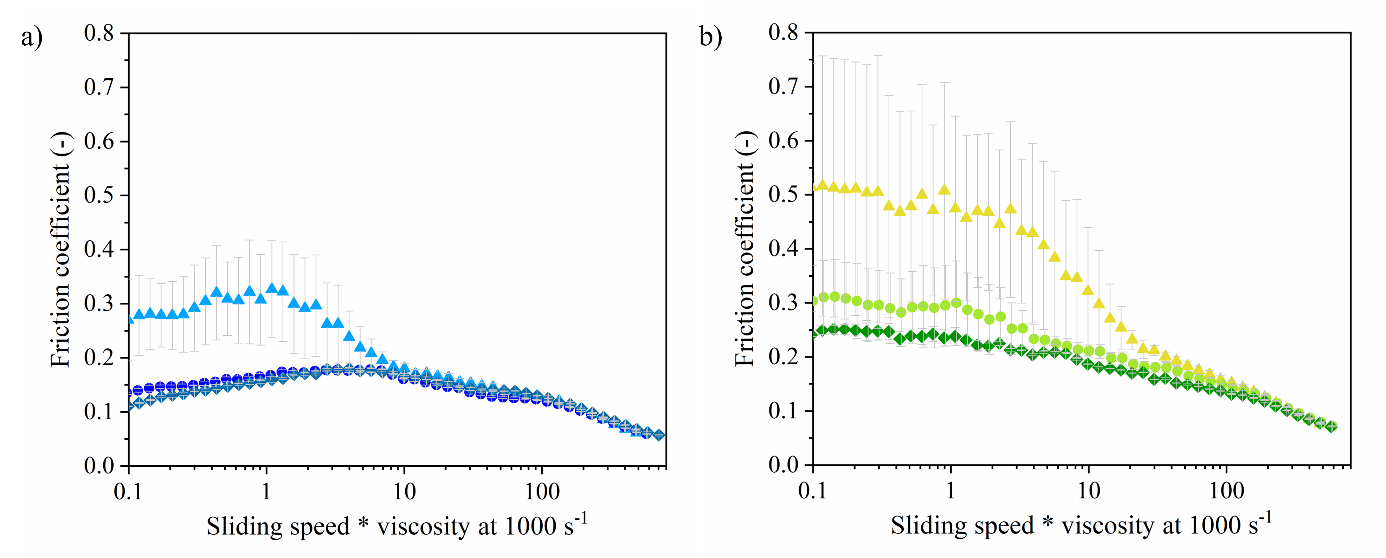


**Figure S3.** Lubrication curve corrected with viscosity at a shear rate of 1000 s^-1^ of a) PPIC-stabilized emulsion at concentrations of 2.5 (light blue triangle), 5 (blue circle) and 7.5% (steel blue diamond); b) PPIL-stabilized emulsion at concentrations of 2.5 (yellow triangle), 5 (light green circle) and 7.5% (green diamond).
